# EHMTI-0359. Migraine pain location and measures of healthcare use and distress: an observational study

**DOI:** 10.1186/1129-2377-15-S1-J8

**Published:** 2014-09-18

**Authors:** E Loder, E Weizenbaum

**Affiliations:** 1 Brigham and Women's Faulkner Hospital, John R. Graham Headache Center, Boston, USA

## Introduction

Previous research suggests that pain arising on the left side of the body may be associated with greater emotional distress and use of healthcare services than right-sided pain. Migraine is a highly prevalent condition in which lateralized pain is a core diagnostic feature.

## Aims

We sought to evaluate whether patients with exclusive or predominant left-sided migraine pain experienced higher levels of distress or healthcare use compared with those with right-sided pain.

## Methods

We extracted medical record information for a random sample of 477 patients with migraine seen in 2011 at the John R. Graham Headache Center. Information was collected on patient demographic characteristics, comorbid affective spectrum and other selected psychiatric diagnoses, ED visits and healthcare contacts. Using a scoring system, we categorized patients as having exclusive, predominant, somewhat or no right or left lateralized pain.

## Results

Of the 228 patients with some degree of lateralized head pain, almost twice as many experienced right vs left lateralized pain (47% vs 29%). With one exception (PTSD), there were no statistically significant differences between the groups in measures of affective spectrum disorder comorbidities or healthcare use.

## Conclusions

Taken as a whole, our data do not support the view that left-sided head pain is more distressing than right-sided pain. Although lateralization of head pain is useful in making a diagnosis of migraine, it does not have additional clincal implications. Additionally, its absence does not rule out a diagnosis of migraine, since in our sample roughly half of patients with migraine had bilateral pain.

No conflict of interest.

